# Stability of formoterol concentration in scalp hair of patients using maintenance formoterol therapy for asthma or COPD (INCTFISH)

**DOI:** 10.3389/fphar.2025.1693712

**Published:** 2025-11-13

**Authors:** E. Sportel, K. Wagelaar, M. Brusse-Keizer, J. van der Palen, H. van Veen, M. Wessels, J. van Boven, D. Touw, K. Movig

**Affiliations:** 1 Department of Clinical Pharmacy, Medisch Spectrum Twente, Enschede, Netherlands; 2 Section Cognition, Data and Education, University of Twente, Enschede, Netherlands; 3 Department of Epidemiology, Medisch Spectrum Twente, Enschede, Netherlands; 4 Health Technology and Services Research, University of Twente, Enschede, Netherlands; 5 Department of Pulmonology, Medisch Spectrum Twente, Enschede, Netherlands; 6 Department of Clinical Pharmacy and Pharmacology, University Medical Center Groningen, University of Groningen, Groningen, Netherlands; 7 Groningen Research Institute for Asthma and COPD (GRIAC), University Medical Center Groningen, University of Groningen, Groningen, Netherlands; 8 Department of Pharmaceutical Analysis, Groningen Research Institute of Pharmacy (GRIP), University of Groningen, Groningen, Netherlands

**Keywords:** hair analysis, formoterol, adherence, smart inhaler, asthma, COPD

## Abstract

**Introduction:**

Hair testing provides a non-invasive, objective method with a long (retrospective) surveillance window for assessing medication adherence. Previous studies have developed a method to analyze formoterol in hair and suggest a possible dose-response relationship. The INCTFISH study aims to assess the stability of formoterol concentrations in hair in patients using formoterol for asthma or COPD maintenance therapy.

**Methods:**

In total, 13 subjects ≥18 years of age, with asthma or COPD, who demonstrated stable to intermediate adherence and medication possession ratio, were included in this pilot study. To study the stability of formoterol, two hair segments located at one and 3 cm from the patient’s scalp were collected, corresponding to a time frame of approximately 3 months. Scalp hair samples were taken during a study visit and analyzed using Liquid Chromatography-Tandem Mass Spectrometry.

**Results:**

Inhaled formoterol was detected in most hair samples with all hair colors in a concentration range of 0.73–7.6 ng/g. The intraclass correlation between segment one and two was excellent (ICC = 0.97).

**Discussion:**

The pilot study reveals a strong correlation between formoterol concentrations in segment one and two, indicating that drug levels remain stable in hair over a period of at least 3 months. While the study does not yet provide sufficient evidence to establish the utility of hair testing for monitoring formoterol adherence, it provides important directions for future research.

## Introduction

Asthma and chronic obstructive pulmonary disease (COPD) are chronic respiratory conditions associated with a high burden of disease and mortality. One of the commonly used treatments for both asthma and COPD is formoterol, a fast and long-acting bronchodilator with predominantly beta-2 effects (VZ info, 2024; Moore et al., 1998; GOLD Report, 2024). While being an effective treatment, both the Global Initiative for Asthma (GINA) and the Global Initiative for Chronic Obstructive Lung Disease (GOLD) emphasize the importance of treatment adherence for optimal patient outcomes (GOLD Report, 2024; GINA, 2024). In addition, GINA states that adherence should be ensured before escalating therapy with a biological drug (GINA, 2024).

For both asthma and COPD, poor therapy adherence has been associated with exacerbations, uncontrolled disease and suboptimal outcomes. Good adherence to inhaled therapy can potentially reduce the risk of exacerbations through symptom control, improving outcomes and reducing healthcare costs (Dekhuijzen et al., 2018; GOLD Report, 2024). Medication adherence in COPD patients is generally poor, varying from 54%–61% for inhaled corticosteroids (± long-acting beta2 agonists) and tiotropium (Koehorst-Ter Huurne et al., 2016). For asthma, up to 60% of patients do not fully adhere to their prescribed medication (Plaza et al., 2021). Current methods for measuring adherence such as patient self-report, pill counts and pharmacy dispensing records are suboptimal, being either prone to recall or social desirability bias and/or being inaccurate in reflecting actual medication intake (Erickson et al., 1998; Zijp et al., 2021; Dierick B. H. J. et al., 2022; World Health Organization, 2025). As such, there is a need for novel methods to determine medication adherence.

Hair analysis, traditionally used in forensic investigations, is gaining interest in pharmaceutical applications such as detecting recreational drug use, (chronic) intoxication and poisoning, therapy compliance control and post mortem toxicology (Paterson et al., 2009; Methling et al., 2020; Zijp et al., 2021). Inhaled medications may enter the systemic circulation via the respiratory and the gastrointestinal tract. From the systemic circulation, a small part of the inhaled drug is transferred to hair tissue as described in detail in a review by Cuperus et al. (2025) The rationale for hair analysis is that drugs can be incorporated from the systemic circulation via the hair vessels into hair during its growth phase (Ramírez Fernández et al., 2020; Kuwayama et al., 2022). However, factors such as hair color, hair type, incorporation via external sources (sweat, sebum, aerosols) and cosmetic treatments can influence drug incorporation into hair, and the extent of these effects remains uncertain (Hassall et al., 2018). Hair testing is a non-invasive procedure offering an objective measure and larger (retrospective) surveillance window (up to 3 months) for medication adherence compared to urine or blood tests. As scalp hair grows approximately 1 cm per month, each centimeter further away from the scalp reflects historic average monthly exposure.^14^

Existing literature has documented the incorporation of corticosteroids and formoterol into human hair in forensic, doping, and clinical case reports, providing a basis for exploring hair analysis as a tool in respiratory pharmacotherapy (Gaillard et al., 2000; Kintz P et al., 2000; Noppe G et al., 2015). Previously, a method for analyzing formoterol in hair has been developed, with some studies suggesting a dose-response relationship (Hassall et al., 2018; Lamy et al., 2020; Dierick et al., 2024). This demonstrates the potential of hair analysis for measuring adherence to formoterol. However, for clinical implementation, further research is needed to explore the dose-response relationship in real-world settings over multiple months. Initially, the stability of formoterol in hair must be confirmed to assess the method’s value for long-term adherence monitoring.

The INCTFISH study aims to assess the stability of formoterol concentration in hair among patients who use formoterol as maintenance therapy for asthma or COPD. Our hypothesis is that formoterol concentrations in scalp hair remain stable over time, providing a reliable biomarker for long-term adherence monitoring.

## Methods

### Study design

A prospective observational analytical proof-of-concept study was conducted with 13 ambulatory asthma or COPD patients from Medisch Spectrum Twente (MST) hospital in the Netherlands, in collaboration with the University Medical Center Groningen for analyzing the hair samples. Recruitment took place from February 2024 through March 2025. The protocol was approved by the medical ethics committee Twente (ID no. K23-40) and conducted in agreement with the Declaration of Helsinki principles. Written informed consent was obtained from all patients before they were included in the study.

### Inclusion and exclusion criteria

Patients were included if they were aged 18 years or older and had been using formoterol as maintenance therapy for at least 12 months prior to inclusion. Eligible patients had to demonstrate stable intermediate or good adherence to their formoterol therapy, as defined by consistent adherence classification according to the validated Test of Adherence to Inhalers (TAI) (Plaza et al., 2016) at two assessment points (t = 0 and t = 1) and a Medication Possession Ratio (MPR) as assessed by electronic pharmacy dispensing records needing to be between >75% and <125% (Huurne et al., 2015). Additionally, patients were required to exhibit an adequate inhalation technique as verified by the researcher.

Patients were excluded from the study if they were unable to speak or understand the Dutch language. Furthermore, patients were excluded if they had insufficient scalp hair length (<3 cm) or if their hair had been cosmetically treated through coloring, perming, or bleaching, as these factors could interfere with the analysis of formoterol concentrations in hair.

### Study procedures

Patients were contacted through referrals by pulmonologists. Screening for eligibility was performed prior to recruitment, focusing on individuals who had been using formoterol as maintenance therapy for at least 1 year.

**FIGURE 1:**
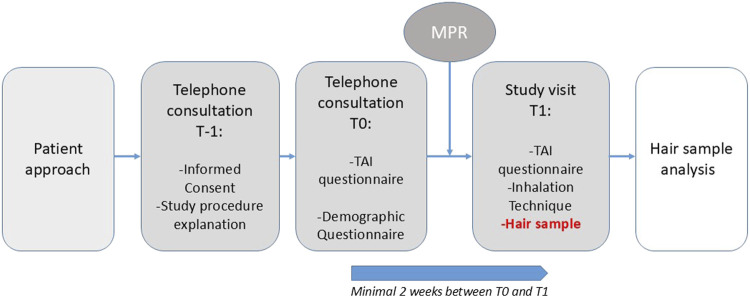
Study procedure (MPR = Medication Possession Ratio, TAI = test of adherence to inhalers).

If interested, patients received detailed verbal and written information about the study, including an information letter and informed consent form (T-1), see Figure 1. Once informed consent was obtained, patients were asked to complete two questionnaires during a structured telephone interview (T0): the Test of Adherence to Inhalers (TAI) and a general questionnaire, concerning demographic information and information about hair (treatment). The follow-up study visit (T1) was scheduled at least 2 weeks after T0.

Prior to the study visit, prescription data for formoterol were obtained from the patient’s community pharmacy. The MPR was calculated by dividing the number of days covered by medication collected from the pharmacy by the expected number of days of use based on physician prescription (e.g., twice daily). Stable adherence was defined as an MPR between >75% (to exclude underuse), and <125% (to exclude overuse) (Huurne et al., 2015). Formoterol is primarily cleared via direct glucuronidation and renal excretion, with only minor involvement of CYP2D6 and no meaningful contribution from CYP2C9. As such, clinically significant pharmacokinetic interactions with inhibitors or inducers of CYP2D6 or CYP2C9 are considered unlikely. Therefore, data on concomitant medications affecting these enzymes were not collected or adjusted for in this study.

Study visits were conducted either at the hospital or at the patient’s home. To minimize the risk of environmental contamination, patients were instructed to wash their hair with their own shampoo and refrain from using hair styling products, such as gel or mousse, day of the study visit. During the follow-up study visit (T1), the TAI questionnaire was administered a second time, and inhalation technique was assessed by the researcher using the inhalation device score list described by van der Kolk et al. and the Dutch guidelines provided at the website of inhalatorgebruik. nl (van der Kolk et al., 2021; Author anonymous, 2022). To assess the stability of formoterol, we collected two hair segments located at one and 3 cm from the patient’s scalp—referred to as segment 1 and segment 2—corresponding to a time frame of approximately 3 months. During visit T1, a hair sample comprising approximately half a pencil thickness) was collected from the posterior vortex of the scalp, as close to the scalp as possible to ensure the inclusion of the most recent hair growth. The hair sample was subsequently divided into two segments of 1 cm each, corresponding to the first and third centimeter from the scalp. Samples were subsequently sent to the Clinical Pharmacy & Pharmacology laboratory of the University Medical Center Groningen (Netherlands) for analysis.

### Chemical analysis of formoterol

Chemical analysis of formoterol was conducted using Conventional Segmental Hair Analysis (Lamy et al., 2020). Quantification of formoterol concentrations was performed using a validated Liquid Chromatography–Tandem Mass Spectrometry (LC-MS/MS) method.

For sample preparation, 20 mg of every hair segment was weighted and washed twice with dichloromethane to remove any external contaminants. After the addition of grinding balls and 1.0 mL methanol containing the internal standard to the dried hair sample, extraction was performed by pulverization for 60 min using a Retsch ball mill at 30 Hz. The samples were centrifuged at 10,000 *g* for 5 minutes and 500 µL of the supernatant was filtered using Whatman mini-uniprep filter vials before LC-MS/MS analysis.

Liquid chromatography was carried out on a Vanquish (Thermo Scientific) system consisting of an autosampler set at 10 °C, a binary pump and a column compartment set at 60 °C. After the injection of 5 µL of the filtered hair extract, formoterol and its stable isotope labeled standard were eluted from an Accucore C_18_ column (2.6 µm particle size, 50 × 2.1 mm; Thermo Scientific) using a gradient of 20 mM ammonium formate buffer (pH 3.5) and methanol with a flow rate of 1.0 mL/min. Detection was performed after heated electrospray ionization with an Altis Plus triple quadrupole mass spectrometer (Thermo Scientific) in positive ion mode.

As part of the method validation and according to FDA (Food and Drug Administration US Department of Health and Human Services, 2018) and EMEA (European Medicines Agency, Committee for Medicinal Products for Human Use, 2022) guidelines selectivity and specificity, linearity, accuracy and precision and stability of processed samples were investigated.

### Statistical analysis

This study was designed as a proof-of-concept study linked to the pilot study conducted by Dierick et al., with a similar study design and methodology (Dierick B. J. H. et al., 2022). Therefore, a sample size of 12 patients was chosen, consistent with the recommended sample sizes for pilot studies (Sim and Lewis, 2012). Statistical analyses were performed using IBM SPSS Statistics version 28 (IBM Corp., Armonk, NY, USA). A p-value below 0.05 was considered statistically significant.

Descriptive statistics were used to summarize patient characteristics. Normality of continuous variables was assessed using histograms and the Shapiro-Wilk test. Normally distributed data were presented as mean ± standard deviation (SD), whereas non-normally distributed data were presented as median and interquartile range (IQR).

The primary endpoint of this study was the stability of formoterol concentrations in hair, by comparing the first with the third centimeter of hair. This was measured using a scatterplot and the intraclass correlation coefficient (ICC) with a two-way random effects model to evaluate absolute agreement between the two hair segments. The reliability of the ICC was assessed following established guidelines: poor (0–0.5), moderate (0.5–0.75), good (0.75–0.9), and excellent (0.9–1). A paired t-test was conducted to evaluate potential systematic differences in formoterol concentrations measured in the two hair segments. Additionally, a sensitivity analysis excluding measurements below the lower limit of quantification (LLOQ) was performed.

## Results

### Inclusion

**FIGURE 2:**
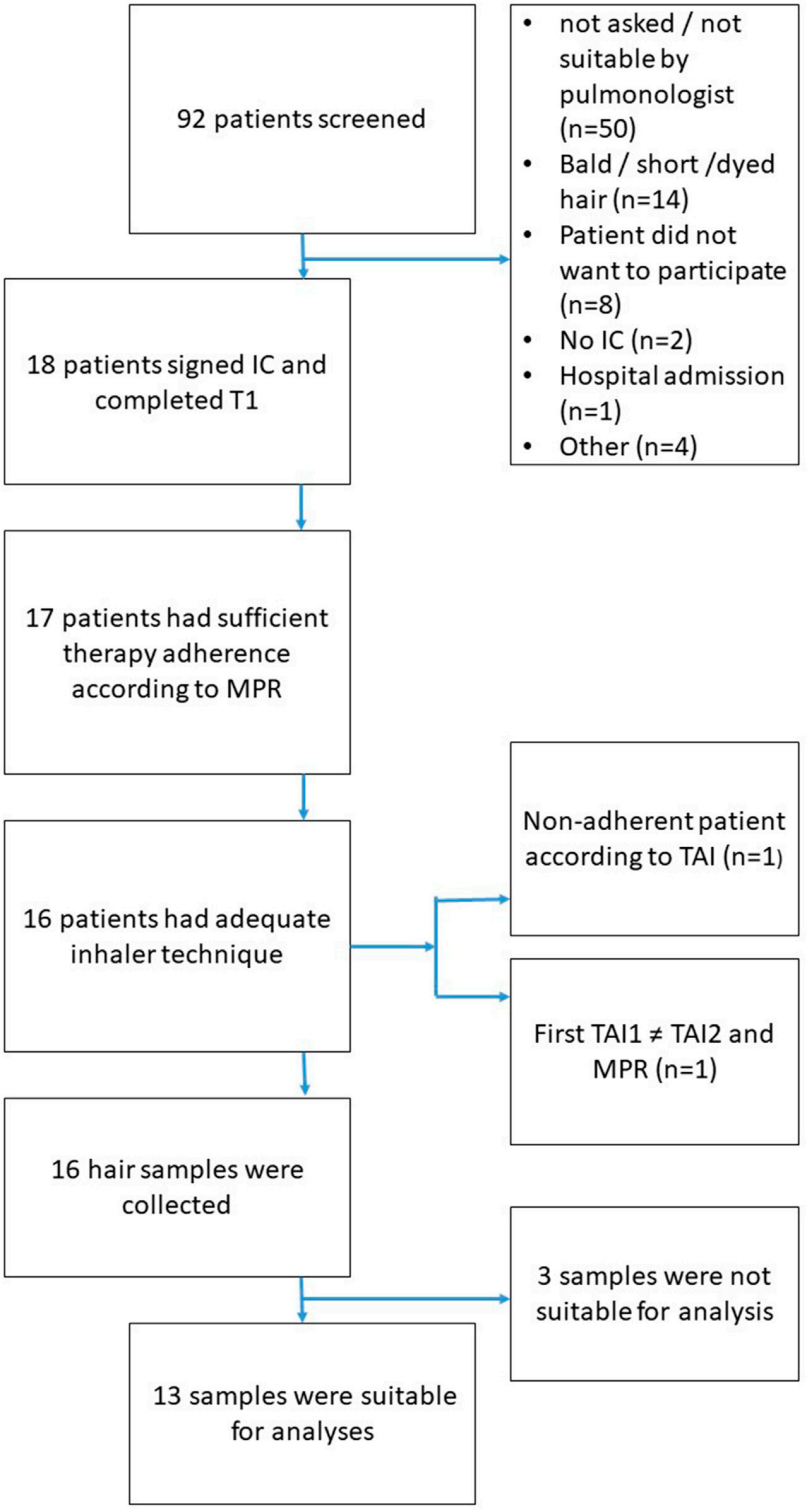
Flowchart of patient inclusion.

Figure 2 illustrates the flowchart of patient inclusion in the study. Out of 92 patients screened, 18 provided informed consent and completed the initial assessment (T0). The most common reasons for exclusion were ineligibility determined by the pulmonologist (n = 50, this could be for any reason, e.g., participation in other studies, limited knowledge of the Dutch language) and unsuitable hair characteristics, such as baldness, very short hair, or dyed hair (n = 14). Additional exclusions occurred due to refusal to participate (n = 8), lack of informed consent (n = 2), recent hospital admission (n = 1), and other miscellaneous reasons (n = 4).

Of the 18 patients who completed the initial assessment, 17 demonstrated sufficient medication adherence based on the MPR. One patient was classified as non-adherent according to the TAI. Additionally, one patient was excluded due to discrepancies between TAI (at T0), TAI (at T1), and MPR, resulting in 16 patients with adequate adherence and inhaler technique eligible for hair sampling. Hair samples were successfully collected from all 16 eligible patients. However, three samples were unsuitable for analysis due to quality issues or insufficient quantity. Consequently, 13 patients were included in the final analysis.

### Subject characteristics and hair treatment

A total of thirteen subjects were included in the analysis, with a mean age of 66.7 years (±11.1), see Table 1. The majority of participants were male (62%), and slightly over one-third (38%) had a diagnosis of asthma. The mean prescribed daily dose (PDD) of formoterol was 22.1 µg (±6.6 µg), with a median MPR of 110% (IQR 96–119) and mean TAI score of 49.9 (±0.4).

**TABLE 1:** Demographic baseline characteristics and hair treatment (N = 13). Mean values ± SD are presented, unless otherwise stated.

Age (years)	66.7 (±11.1)
Gender (male %)	8 (62)
Indication asthma/COPD (asthma %)	5 (38)
Formoterol daily defined dose (µg)	22.1 (±6.7)
Medication possession ratio % (median, IQR)	110 (96–119)
Test of adherence to Inhalers score (10-items)	49.9 (±0.4)
Hair color	
Grey (%)	6 (46)
Blond (%)	4 (31)
Dark (%)	3 (23)
Hair treatment	
Conditioner (%)	1 (8)
Hair gel/mousse/spray (%)	6 (46)
Hair serum/oil (%)	1 (8)

Regarding hair characteristics, grey hair was the most common, observed in 46% of subjects, followed by blond hair (31%) and dark hair (23%).

Hair treatment practices varied among participants. All subjects reported the use of shampoo, while only one person used conditioner. More than half of the participants (54%) applied styling products during the use of inhalation medication.

### Formoterol concentration analysis

The developed analytical method resulted in a total runtime of 1.6 min with formoterol and its stable isotope labeled standard having a retention time of 0.7 min. Both compounds were detected as the most intense fragment of the [M + H]^+^-adducts with *m/z* = 345.2→149.1 and *m/z* = 349.3→153.1 respectively.

Selectivity and specificity was one of the parameters evaluated during the validation of the LC-MS/MS method. Six different lots of blank hair including different hair color, dyed and non-dyed and from male as well as female donor, were processed without the addition of formoterol or its internal standard. The attribution of possible interfering compounds, was found to be less than 14.4% of the peak height of formoterol at LLOQ level and not more than 0.3% of the response of its internal standard. Method validation showed good linearity (weighing 1/x) over a concentration range of 0.8–50 ng formoterol per gram hair. Accuracy and precision were determined in fivefold and on three different days at quality control (QC) levels LLOQ (0.8 ng/g), Low (2.0 ng/g), Medium (10 ng/g) and High (40 ng/g). This resulted in accuracies of 104.2%–114.6% at LLOQ level and 91.2%–109.6% for QCs Low, Medium and High. Within-run, between-run and overall precision, expressed as correlation of variance, were less than 4.1%, less than 5.9% and less than 6.2% respectively. Stability of processed samples was investigated by reinjecting the QC Low and QC High samples of day one of the validation with the runs of the other validation days. The peak height ratios of formoterol and its internal standard of the reinjections have been compared to those of day one (t = 0). After storing the processed samples in the autosampler (at 10 °C) for 15 days, the peak height ratios of QCs Low and QCs High were 96.7% and 98.5% of the ratios at t = 0 respectively. The results off all evaluated validation parameters met the criteria of the FDA and EMEA guidelines.

Table 2 summarizes the formoterol concentrations in micrograms per gram hair (ng/g) measured in the hair samples, divided into two hair segments (segment 1 and segment 2, on 1 respectively 3 cm from the scalp). The table also provides information on hair color, gender, and hair treatment and PDD for each subject. Formoterol was below the LLOQ (0.8 ng/g for 20 mg of hair) in both segments for four subjects. The subject with the highest concentrations of formoterol (7.4 ng/g in segment 1 and 7.6 ng/g in segment 2), reported the use of no styling products and had blond hair.

**TABLE 2:** Concentration formoterol per hair segment with possibly influencing factors grouped by hair color (*<LLOQ* = below lower limit of quantification).

Subject	Formoterol concentration (ng/g) segment 1 (1 cm from scalp)	Formoterol concentration (ng/g) segment 2 (3 cm from scalp)	Prescribed daily dose formoterol (µg)	Hair color	Gender	Hair treatment
1	0.85	0.73	12	Blond	m	—
2	*<LLOQ*	*<LLOQ*	24	Grey	m	—
3	7.4	7.6	24	Blond	m	—
4	1.2	0.80	24	Grey	f	Mousse
5	3.8	5.2	24	Dark	m	Gel, mousse
6	1.9	**2.5**	24	Grey	f	Gel
7	*<LLOQ*	*<LLOQ*	24	Blond	m	
8	*<LLOQ*	1.0	24	Blond	m	—
9	2.2	2.3	24	Grey	m	Gel
10	2.0	2.1	24	Grey	f	Spray, serum
11	*<LLOQ*	0.77	36	Grey	m	Gel
12	*<LLOQ*	*<LLOQ*	12	Dark	f	—
13	*<LLOQ*	*<LLOQ*	12	Dark	f	—

Subjects with grey hair who used styling products (e.g., mousse, gel, spray, serum) tended to have detectable formoterol levels, while the untreated hair from one grey-haired subject showed no detectable levels. The same pattern was detectable for dark hair. For subjects with blond hair, there was no pattern to be detected between hair treated or not with styling products, both hair samples showed detectable and <LLOQ levels of formoterol. Gender did not appear to influence the detectability of formoterol.

### Correlation between formoterol concentrations in hair segments

**FIGURE 3:**
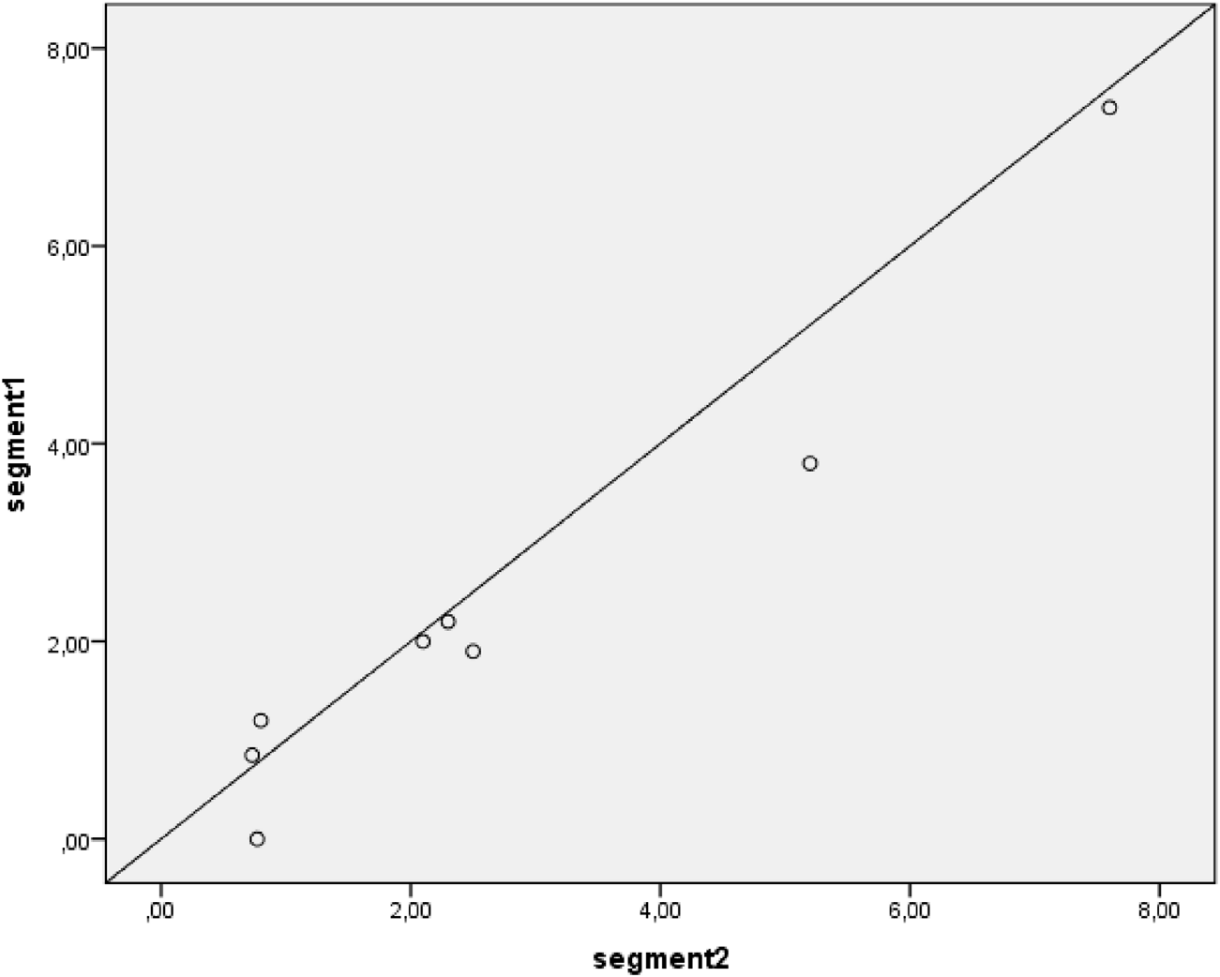
Correlation between segment 1 and segment 2 hair concentrations of formoterol. Including all available samples; samples with results below Lower Limit of Quantification (LLOQ) are plotted as 0 ng/g. The diagonal line depicts a perfect intraclass correlation.

**FIGURE 4:**
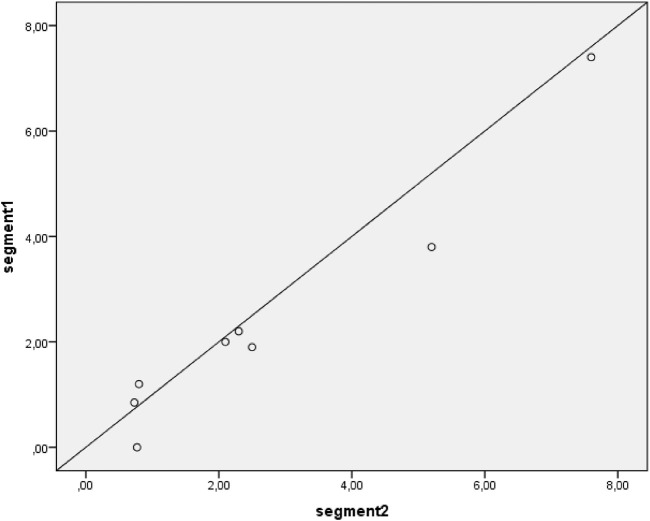
Correlation between segment 1 and segment 2 hair concentration of formoterol. Samples with results below Lower Limit of Quantification are not plotted. The diagonal line depicts a perfect intraclass correlation.

The scatter plot presented in Figure 3 shows an excellent intraclass correlation between the two segments (ICC = 0.97, p < 0.001). For a sensitivity analysis, the measurements below the lower limit of quantification are not plotted (Figure 4), which showed a similar intraclass correlation between the two segments (ICC = 0.97, p < 0.001).

## Discussion

The data demonstrated a strong association between formoterol concentrations in hair segments at one and 3 cm, suggesting that formoterol levels remain stable in hair over a period of at least 3 months. This stability indicates that the measurement method is both reproducible and precise, although its external validity warrants further investigation. The high intraclass correlation coefficient (ICC) underscores the consistency of measurements across segments. Notably, the ICC remained high even after performing sensitivity analyses, highlighting the robustness and reliability of the method. It reinforces the potential value of hair analysis for assessing long-term patient exposure, as previously proposed by Salvator et al. (2023).

While our results confirm high internal validity (within patients), external validity (between patients) cannot be established given the variation in concentrations across patients. Indeed, the presence and concentration of formoterol in hair may also be influenced by external factors such as hair styling type and the frequency of such treatments. Our results suggest that the use of hair styling products such as gel and spray positively influence the binding of formoterol in hair samples in grey and dark hair; The question is whether the hair care products promote the incorporation of formoterol into the hair, or whether these products protect formoterol from external environmental factors. This finding is particularly concerning, as it suggests that external cosmetic factors could falsely elevate measured drug levels. This introduces the risk of misclassifying a non-adherent patient as adherent simply due to hair product use during a given period—possibly undermining the reliability of hair analysis as an objective measure of adherence. These external and physiological factors should be systematically addressed in future study designs to strengthen the robustness and generalizability of hair-based adherence monitoring (Cuperus et al., 2025).

Although preliminary data suggest a potential relationship, further analysis is required to confirm these findings and quantify their impact. Both the chemical composition of the different cosmetic agents and the nature of the methods applied in cosmetic treatments affect drug stability (Barbosa et al., 2013). In literature, we see a similar line of thought, but definitive conclusions remain tentative (Hassall et al., 2018). Hair treatments such as dyeing and especially bleaching is known to have a large impact on the concentrations of drugs in hair, so this was an exclusion factor for our study for the correct interpretation of the results (Jurado, 1997; Van Elsué and Yegles, 2019).

Beyond formoterol, various other pharmacological agents can be measured in hair. We know that during the COVID-19 pandemic, an increased interest in remote monitoring demanded reliable and non-invasive methods for patients to provide samples from their homes (Capiau et al., 2019). In this study, the majority of participants used a 24-μg dose of formoterol, which limited our ability to detect a correlation between the PDD of formoterol and its concentration in the first or third centimeter of hair. Although previous studies have reported such an association, our data could not confirm it. After excluding extreme outliers, false positives, and undetectable results, Hassall reported a strong correlation coefficient of 0.964 for formoterol, primarily in asthmatic individuals with dark hair (Hassall et al., 2018). Methling et al. (2020) analyzed concentrations of antidepressants and antipsychotics in hair (Methling et al., 2020), while Ferrari demonstrated the potential of hair analysis as a reliable marker of adherence in headache treatments (Ferrari et al., 2017). Similarly, Takiguchi highlighted the value of hair analysis for assessing individual drug-taking behavior with flecainide (Takiguchi et al., 2002).

### Limitations

Our study has several strengths, to ensure the reliability and robustness of the results, all samples in our study were included in the analysis and obtained from patients with confirmed medication adherence and correct inhaler technique, thereby strengthening the validity of the measured hair concentrations, although we had a small sample size. By conducting a sensitivity analysis, we demonstrated the robustness of our findings. The study population included subjects with varying hair colors and diagnoses of both asthma and COPD, as well as differences in hair treatment practices, thereby representing the average real-world population.

Some limitations of this study should be acknowledged. The assertion that formoterol is stable in hair is based on a limited sample size, in which approximately 1/3 of the participants had undetectable or unquantifiable levels of the drug. Values below the limit of quantification were imputed as zero, which may have affected the accuracy of low-concentration readings. Although it was not possible to determine the underlying cause of the low concentration in hair, one of our main assumptions was that extracting the prescribed quantity of hair directly from the scalp is essential for reliable analysis. In certain hair samples, the lower limit of quantification (LLOQ) threshold was elevated due to the insufficient amount of hair sample extracted. These elevated LLOQ adjustments may introduced a degree of bias in the correlation analysis, probably making the overall correlation appear stronger than it is and resulted in a higher proportion of participants with drug levels falling below the lower limit of quantification (LLOQ). We performed a sensitivity analysis to check whether coding values below the LLOQ as zero had any direct impact on the correlation.

To enable a more detailed analysis of the correlation between PDD of formoterol and its concentration in hair, a larger sample size is required, stratified by hair color, use of hair styling products, and a broader range of prescribed daily doses. As this was a pilot study involving only 13 subjects, the sample size was insufficient to draw conclusions regarding these associations.

While MPR and TAI are established and validated measures of medication adherence, their dependence on prescription refill data and patient self-reporting introduces inherent subjectivity and limits their objectivity. In the present study, participants were informed of the study objectives, which may have fostered accurate self-reporting among individuals who perceived themselves as adherent. Nevertheless, we found approximately undetectable or unquantifiable levels of formoterol in 1/3 of the participants, suggesting either non-adherence or poor inhalation technique besides e.g., deviations from the hair sampling protocol. We believe that the integration of objective inhaler-use data could strengthen the accuracy of associations with pharmacokinetic biomarkers, including the determination of formoterol levels in hair. Future investigations could benefit from incorporating smart inhaler technologies, which offer real-time, objective monitoring of both adherence and inhalation technique, when measuring formoterol in hair in a larger, more diverse sample (Cuperus et al., 2024).

## Conclusion

This pilot study demonstrates a strong correlation between formoterol concentrations in hair segments at one and two, indicating that drug levels remain stable in hair over a period of at least 3 months. These exploratory findings support the reproducibility and analytical precision of this non-invasive, retrospective monitoring method. While the study does not yet provide sufficient evidence to establish the utility of hair testing for monitoring formoterol adherence, it provides important directions for future research.

## Data Availability

The raw data supporting the conclusions of this article will be made available by the authors, without undue reservation.
